# Exploring the Impacts of Environmental Factors on Adolescents’ Daily Participation: A Structural Equation Modelling Approach

**DOI:** 10.3390/ijerph18010142

**Published:** 2020-12-28

**Authors:** Yael Fogel, Naomi Josman, Sara Rosenblum

**Affiliations:** 1 Department of Occupational Therapy, School of Health Sciences, University of Ariel, 40700 Ariel, Israel; 2 The Laboratory of Complex Human Activity and Participation (CHAP), Department of Occupational Therapy, Faculty of Welfare and Health Sciences, University of Haifa, 3498838 Mount Carmel, Israel; naomij@research.haifa.ac.il (N.J.); rosens@research.haifa.ac.il (S.R.)

**Keywords:** daily activities performance, executive function deficit (EFD), home, school, community, supportive factor, structural equation modelling

## Abstract

Adolescents with neurodevelopmental difficulties struggle to perform daily activities, reflecting the significant impact of executive functions on their participation. This research examines an integrated conceptual model wherein supportive environmental factors in the community, school and home settings explain the children’s participation (involvement and frequency) with their daily activities performance as a mediator. Parents of 81 10- to 14-year-old adolescents with and without executive function deficit profiles completed the Participation and Environment Measure for Children and Youth and the Child Evaluation Checklist. A secondary analysis was conducted to examine the structural equation model using AMOS software. The results demonstrated support for the hypothesised model. Supportive environmental demands in school predicted 32% of home participation, and the adolescents’ daily performance reflected that executive functions mediated the relationship between them. Together, these findings highlight the school environment as the primary contributor that affects the children’s functioning according to their parents’ reports and as a predictor of high participation at home in terms of frequency and involvement. This study has implications for multidisciplinary practitioners working with adolescents in general, and in the school setting specifically, to understand meaningful effects of executive functions on adolescents’ daily functioning and to provide accurate assistance and intervention.

## 1. Introduction

Participation in daily activities naturally occurs when individuals involve themselves in occupations (daily life activities) that have significance and purpose [1]. Within contemporary theory, participation results from the dynamic transactions between an individual and their environment [2]. The World Health Organization’s [3] International Classification of Functioning, Disability and Health: Child and Youth (ICF-CY) version also demonstrated that personal and environmental factors affect interactions among body structure and function, performing daily activities and participating in the community. Adolescents who participate in daily activities form strong bonds with their communities and develop their roles in society, which then helps them prepare for adulthood [4].

Since the ICF-CY was developed, there has been a continued effort to refine the understanding of participation and environmental factors that support or inhibit children both with and without disabilities over time [5]. Maciver et al. [6] reviewed the association among environmental and psychosocial factors with participation in school of children aged 4 to 12 years. Their findings supported the hypothesis that participation outcomes are influenced by known contexts and mechanisms. Specifically, Maciver et al. showed that school routines and structures, objects and spaces and peers and adults are representations of the environment (context). Concerning identified mechanisms, Fogel et al. [7] showed that children with executive function deficits (EFD) faced more barrier factors in the environment than did their peers and found the activities’ social and cognitive demands to be the most challenging.

Executive functions (EFs) are a neuropsychological concept referring to a skillset that composes the cognitive process. This skillset allows people to forsake immediate demands to instead achieve long-term goals and thus to organise their behaviour over time [8]. These EFs influence participation and performance in daily life [9], and the performance of most daily activities requires using different EF components. The literature indicated that EFs might serve as an underlying mechanism in neurodevelopmental disorders such as attention deficit hyperactive disorder (ADHD), specific learning disorder and developmental coordination disorder. The contribution of EFs to adolescents’ participation [7], scholastic achievements [10] and daily functioning has been reported [11]. The transition from childhood into adolescence often brings a new set of responsibilities and self-regulatory requirements (e.g., in school and social environments) [12] that necessitate adolescents to rely more on this emerging cognitive control.

Recently, Fogel et al. [13] described adolescents with EFD profiles. These adolescents are characterised as impaired when performing complex daily living activities. They often struggle to achieve everyday life goals as efficiently as their peers without EFD. That is, they require considerably more help from adults, need substantially more time to complete tasks and exhibit behaviours that are far more dangerous [13]. Since adolescents with EFD profiles tend to focus on immediate timeframes, they find planning to be a challenge. They also struggle to shift between activities, prioritise essential tasks, manage their time and meet deadlines [14]. These difficulties hinder their effective participation and performance in everyday life, creating a functioning gap between them and adolescents without EFD [11,13,15].

In the existing literature, discussion of the relationships among performance of daily activities, environmental factors and participation is scarce, and the overall picture—including clinical implications—is still unclear. Noreau and Boschen [16] dealt with the complex environment of participation interaction. Their results indicated that despite the environment’s obvious theoretical impact on participation, its contribution to restricting or facilitating participation has yet to be demonstrated scientifically. King et al. [17] reported the environment’s indirect impact on participation by referring to its direct effects. Specifically, their results showed the adolescent’s activity preferences and functional abilities, as well as the family’s orientations, to be the most important predictors of participation. Moreover, they indicated the need for a more in-depth look at indirect effects to broaden viewpoints and to consider the roles that other environmental and family factors play in what had been presumed to be causal, developmental sequences.

In contrast, Anaby et al. [18] found that the environment played a mediating role. Their findings explained the participation of young children with or without disabilities across community, school and home settings. Anaby et al. proposed and tested one model for each setting using structural equation modelling (SEM). These models explained 50% to 64% of the variance in both involvement and frequency of participation. According to these results, supports and barriers in the environment significantly mediate between the adolescent’s personal factors (e.g., health and functional issues or income) and participation outcomes.

Likewise, most other studies on participation showed that children and adolescents with disabilities participate less in daily activities in terms of level of involvement and frequency in all three settings [19,20,21] and face more inhibiting environmental factors [7,18]. However, questions about the impact of the child’s everyday expression of daily activities performance, and how it relates to participation, are still unanswered and need additional research.

This lack of a documented, compelling association between participation and environmental factors denotes how difficult it is to operationalise these constructs [16]. Therefore, this study examines the extent to which factors that support adolescents’ environment also influence their participation. It assumes the mediating factor is adolescents’ daily activities performance. To that end, this research uses the Participation and Environment Measure for Children and Youth (PEM-CY) [20,22], which is a reliable, valid and well-documented tool for assessing both participation and environmental factors. Additionally, the study uses the Child Evaluation Checklist (CHECK) questionnaire, which was also found to be valid and reliable, to examine the daily activities performance that reflects EF in young children [23,24] and adolescents [25]. Combining these two questionnaires (completed by the adolescents’ parents) connects the current concept presented by the ICF-CY [3], which views children’s and adolescents’ functioning holistically. It also reflects previous studies’ recommendations to examine the complexity of the relationship between participation and environmental requirements and abilities.

**Figure 1:**
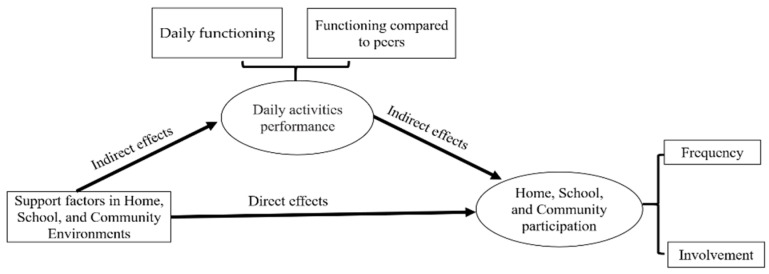
Conceptual model.

This study assumes that support factors in all three environments (community, school and home) may improve adolescents’ daily activities performance and thus affect their participation (involvement and frequency) in the various environments. Figure 1 depicts the proposed theoretical model underlying the direct and indirect factors impacting participation. This conceptual model assumes that if the environment is supportive, then the adolescent’s daily activities performance will be better and thus will affect their participation.

## 2. Materials and Methods

### 2.1. Participants

This study refers to a secondary analysis using data from a previously published study, which detailed the participant inclusion and exclusion criteria [7,13]. In the current study, the data refer to all participants as one group with no separation between adolescents with and without EFD profiles. Specifically, the participants were 81 early adolescents, 10 to 14 years old (*M* = 12.07 years, *SD* = 1.17). Of them, 57 (70.4%) were boys and 24 (29.6%) were girls. In the original study, 41 participants presented with EFD profiles and 40 with typical development (i.e., without EFD profiles). The EFD profiles were defined using the Behavior Rating Inventory of Executive Function (BRIEF) parent [26] and self-reports [27] and WebNeuro assessments [28]. The parents of all 81 adolescent respondents were invited to participate in the study.

### 2.2. Procedure

The University of Haifa Ethics Committee approved this study. Both the parents and the participating adolescents signed informed consent forms. Once accepted into the study, the parents completed a demographic questionnaire. The CHECK provided data regarding the daily activities performance reflecting EF and the PEM-CY as the outcome measure.

We tested two proposed theoretical models. Only one SEM fit the data well and successfully tested both the direct and mediated effects of environmental support factors as an observed (measurable) variable in the community, school and home on the theoretical latent variable, participation. It identified the level of involvement and frequency (10 indicators/items for community, 5 for school, 10 for home) as observed variables, as well as the theoretical latent variable, daily activities performance (consisting of daily functioning and functioning compared to peers).

### 2.3. Measurement Instruments

#### 2.3.1. Demographic Questionnaire

Parents completed the demographic questionnaire, providing data on their education and socioeconomics and on the adolescents’ age and gender.

#### 2.3.2. Child Evaluation Checklist

A brief screening instrument used to identify children at risk for under-recognised, invisible neurodevelopmental conditions, the Child Evaluation Checklist (CHECK) [24], emphasises small nuances in the performance features of children’s daily activities as related to the children’s EFs. The CHECK tool includes two parts. The CHECK-A addresses the current level of daily activities performance, especially frequency. Respondents rate agreement with 30 statements on a Likert scale that ranges from 1 (never) to 4 (always). For example, the statements address whether the adolescents properly estimate the task difficulty and whether they complete tasks they take upon themselves. After exploratory factor analysis, four factors were obtained: organisation (body, essentials and social), self-regulation, performance/expression management and activities of daily living. Cumulatively, these four factors produced a 54.05 variance percentage and α = 94 internal consistency.

The CHECK-B compares the adolescents’ general daily function to peers. Using ranks from 1 (low) to 5 (high), parents respond to statements that contain phrases such as, “Compared to other children, my child …” or “In work habits, my child’s overall functioning is …”.

We calculated an average score for each part and determined internal consistency (CHECK-A, α = 0.96; CHECK-B, α = 0.94). Construct validity was established and documented in [11]. 

#### 2.3.3. Participation and Environment Measure for Children and Youth

Although parents completed the primary outcome measure, the Participation and Environment Measure for Children and Youth (PEM-CY) [20,22], herein we present the results in terms of the adolescents as the “participants”. Part A of the PEM-CY includes 25 items focusing on participation in a diverse range of activities in community (10 items), school (five items) and home (10 items) settings. For each item, parents report the child’s participation through three dimensions: (a) level of involvement on a 5-point scale from 1 (minimally involved) to 5 (very involved); (b) participation frequency on an 8-point scale from 0 (never) to 7 (daily); and (c) parents’ desire for a change (e.g., in either involvement or frequency) of their child’s participation (yes or no). If parents respond yes to a desire for change, they then select whether they want that change in the child’s level of involvement or frequency or a wider variety of activities. However, this study did not include the parents’ desire for change.

In this study, participation level of involvement and frequency were calculated as the average of all ratings, except those (either involvement or frequency) for which the parent answered never. The PEM-CY’s summary score internal consistency for both participation involvement (α = 0.72–0.83) and frequency (α = 0.59–0.70) was moderate to good. Test–retest reliability for all participation and environment summary scores (interclass correlation (ICC) from 0.58 to 0.95) and across items within the instrument’s community, school and home sections (ICC = 0.68–0.96) was also reported as moderate to good [29].

The PEM-CY’s Part B asked parents if specific environmental features aided or hindered their children’s participation in activities in each (community, school or home) setting. When parents reported a feature as an aid, we coded that item as a support factor. If parents reported the feature made things harder (sometimes or usually), then we coded the item as a barrier factor. The PEM-CY’s summary scores internal consistency for both participation involvement (α = 0.72–0.83) and frequency (α = 0.59–0.70) was moderate to good. Test–retest reliability was reported for all participation and environment summary scores (ICC α = 0.58–0.95) and across items within the instrument’s community, school and home sections (α = 0.68–0.96) as moderate to good [22].

### 2.4. Data Analysis

Using the bootstrapping method, SEM was conducted to examine the mediation model. The bootstrapping procedure’s value lies in its ability to process repeated simulations of subsamples from an original database. With this, we could assess the parameter estimate stability and report their values with increased accuracy. Bootstrapping estimates each resampled dataset’s indirect effects and determines a confidence interval for these specific indirect effects [30,31]. We analysed the data using SPSS (version 25) and AMOS software. Indices to evaluate the model included chi-square (acceptable when the value is not significant); comparative fit (CFI); non-normed fit (NNFI; adequate values > 0.90 and excellent fit > 0.95); root-mean-square error of approximation (RMSEA; adequate values < 0.08 and excellent fit < 0.06); and standardised root-mean-square residual (SRMR; <0.08) [32]. Level of significance (*p* value) was 5%.

## 3. Results

Table 1 presents descriptive statistics and Pearson correlations among the study variables. Results show that significant correlations were found between the participation variables (involvement and frequency) as measured by the PEM-CY and daily activities performance reflecting EF as measured by the CHECK (*r* = 0.22–0.88; *p* < 0.050 to *p* < 0.001). 

**Figure 2:**
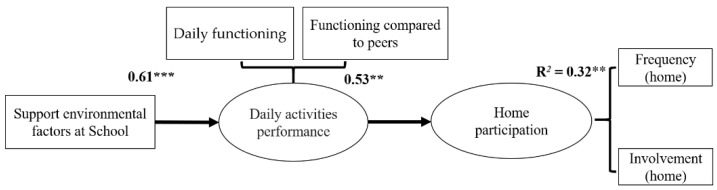
Analysis results of conceptual model mediation. Coefficients in bold are significant at *p* < 0.05. ** *p* < 0.01, *** *p* < 0.001.

The SEM provided excellent goodness of fit indices (χ^2^(21) = 32.08; *p* > 0.05; NFI = 0.95; CFI = 0.98; RMSEA = 0.08; SRMR = 0.08). As depicted in Figure 2, results of this model showed that higher support of environmental demands at school leads to higher daily activities performance (β = 0.61, *p* < 0.001) and relates positively with home participation (β = 0.53, *p* < 0.05). The indirect effect found between support from the school environment and home participation (β = 0.32, *p* < 0.01) means that the daily activities performance is a mediator between environmental demands at school and home participation. The model explained 58% of home participation.

Table 2 supports Figure 2. It represents the regression coefficients among all model components to describe the size and direction of the relationship between a predictor and the response variable.

## 4. Discussion

This study examined the effects of supportive environment factors upon participation among adolescents with and without EFD through an SEM approach. Applying SEM allowed us to isolate both the direct and indirect paths by which the environmental (community, school and home) settings affect the adolescents’ participation across the three settings. Specifically, our results show that supportive environmental factors in school have indirect effects on home participation, while the adolescents’ daily activities performance serves as a mediator of this relationship. That is, no *direct* connection was found between environment and participation; rather, they are connected through the adolescents’ daily activities performance. These results may indicate that as long as there is no improvement in the adolescents’ daily activities performance following the supports they receive at school, we cannot expect a change in the home environment—not in leisure activities, household chores, school preparation or homework.

### 4.1. Supportive School Environment Demands

Role performance in the complex high school environment is critical for academic success; poor role performance in academic and social participation creates high risk for student dropout [33]. The school environment can also often create one of the greatest perceived barriers [34,35]. Unlike several prior studies that described participation barriers without considering the environment’s facilitating aspects [16], this study focused on supportive factors. According to the current findings, a supportive school setting can encourage students with and without EFD to more effectively express their daily activities performance and increase participation in the home environment. For instance, Wehlage [36] gathered information from 14 secondary schools that were selected based on their successful dropout prevention programmes. His key findings were relevant to the current study’s findings. The results suggested that successful schools create a supportive environment that helps students overcome impediments to membership and engagement. Successful programmes matched students’ needs and problems and took advantage of students’ interests and strengths. Recently, Mann and Snover [15] argued that to maximise role performance, environmental influence should be viewed as a means to scaffold and develop EF skills. They mentioned the school environments of interest, including administrative and classroom policies, especially regarding their effects on the interplay between person and role performance (both for students and teachers).

Students and teachers’ social interactions, as influenced by educational and social values, create the school climate [37]. Increasingly, research has documented the association of prosocial and academic motivation, conflict resolution, altruistic behaviours and self-esteem with positive school climates. As do teacher–student interactions, the social and educational values that influence children’s psychological, social and cognitive development also affect the school climate [38]. Such values include the physical environment [39] and safety [40].

In a previous study, Fogel and colleagues [7] highlighted the environmental factors that, according to parents’ reports, best predicted adolescents with EFD. For the school environment, these factors include the activity’s cognitive and social demands and staff and teachers’ attitudes. Fogel et al. found these factors to substantially aid classification of the study population characteristics (i.e., with or without EFD) and prediction of participants’ daily functioning. Since adolescents spend a significant amount of time in school, school activities constitute a significant part of their daily routine both academically and socially.

According to the U.S. Department of Education’s [41] Safe and Supportive Schools model, the school climate includes three interrelated domains: (a) the school environment (disciplinary, wellness, physical and academic), (b) student engagement (school participation, respect for diversity and relationships) and (c) safety (substance use/abuse, physical and social-emotional). Bradshaw et al. [37] explored this model. Their findings added to the growing research regarding associations between student outcomes and school climates, indicating that the school climate can significantly predict student achievement.

In 1935, Lewin [42] studied environmental influences on people’s (especially children’s) behaviour. He suggested that all elements of a child’s behaviour, such as the environment where the child lives or how the child plays, influence the child’s voluntary behaviour and emotions. Lewin expressed that relationships influence which behaviours a child exhibits, and that those behaviours equate to the child’s function and environment.

### 4.2. Daily Activities Performance Reflecting EF

Executive dysfunction may be one among many contributors to difficulties adolescents experience [43]. Impaired EFs can lead to compromised self-regulation and decision making, as well as difficulty performing complex or novel tasks. This, in turn, can negatively affect academic performance across the adolescents’ life span, leading them to become frustrated when their efforts prove ineffectual and unsuccessful and the outcomes are unsatisfactory (e.g., [44,45]). For example, Mann and Snover [15] measured academic performance to examine how EFs can affect students’ role performance and found a significant correlation between poor executive functioning and low academic performance, regardless of setting.

Due to the complexity of recognising such difficulties, children with EFD are most often perceived as having behavioural problems, lazy, lacking motivation, manipulating, “doing it on purpose” and other misleading negative descriptors [9]. Unlike cases of cerebral palsy or intellectual disabilities, for example, the condition of adolescents with EFD is not as clear cut—it may seem invisible. There is a discrepancy between what others can see and what is really happening to these individuals. There are no physical signs, and the adolescents have average or above average intelligence. Nevertheless, the children and their families sense “something different than other children” but do not know what it is or why it is occurring [11]. Unrecognised EFD can compound these effects on daily occupational performance, which then can create secondary issues [46]. Thus, adolescents with EFD must be viewed through the expression of their daily activities performance—seen past their externalising behaviours to understand their daily functioning and recognise their specific needs. Additionally, adaptive programmes and interventions to promote their participation must be created.

### 4.3. Home Participation

Adolescence is marked by increased autonomy and access to adult activities and decreased dependence on primary individuals (e.g., parents) and organisational supports [47]. Despite the natural processes occurring in adolescence, this study’s results show that the model explained 58% of specific at-home setting participation. Previous studies have identified the environment where the child lives and develops [48] as a critical context in which EFs develop [49], suggesting that individual differences in EFs are also associated with the home environment. Typically, this home environment is measured by the nature, frequency and amount of activities parents create for their children to learn [50]. However, few studies have dealt with the relationship between participation in the home setting and EFs among young children. Korucu et al. [51] investigated potential associations among general parenting practices, EF-related activities in the home and children’s EFs beyond the home environment. They discussed the potential importance for pre-schoolers to be exposed in the home environment to EF-specific activities. In 2020, Korucu et al. [52] demonstrated a positive association between more enriching home literacy environments with pre-schoolers’ EFs, which then relate to mathematic skills and readiness for general academics. 

According to the PEM-CY, this home participation includes leisure and play activities, such as video or computer games, indoor games and play, arts and crafts and other hobbies, listening to music or watching TV, and activities that require social interaction, including getting together with others. Activities such as school preparation (e.g., gathering and packing materials, school bags and lunches or reviewing schedules) and doing homework (e.g., assignments, readings and projects) are also included. This is illustrated through a homework example. The process to finish homework assignments is multifaceted. To successfully complete an assignment, the student must initially record it accurately, bring home the materials needed (e.g., textbooks, handouts), allot after-school time to work on (and ultimately complete) the project, possess the skills needed to finish the work and then bring the finished assignment back to school and turn it in. For assignments that require long-term planning (e.g., long-term projects or preparing for exams), that process becomes even more complex [53]. Such typical assignments can overload the weakened EFs of children with disabilities. To finish a homework assignment, students must (a) keep their attention on the task at hand, (b) ignore distractions, (c) make a plan and set objectives, (d) decide on milestones, such as “where to start” and when to complete, (e) consider details as well as the big picture and (f) organise the relevant materials [54]. Children and adolescents with EFD profiles struggle with those kinds of daily activities, similar to previous findings among students with learning disabilities [55]. For instance, Langberg et al.’s [53] findings suggested that the latter task—organising materials—is critical for students with ADHD in their process to complete homework and thus should be prioritised in interventions.

### 4.4. Limitations and Future Studies

Despite its important results, this study has limitations. It included only a small sample in a narrow age range. Larger samples with broader age ranges among adolescents with different disabilities might have expanded the information about the relationship between participation and environment factors across settings. Further, this research did not address parental attitudes towards their children’s daily functioning, all factors that may affect functioning perspectives or perspectives other than the parents’. Future studies might incorporate the adolescents’ perspectives about their daily activities performance, participation and environmental factors. Future research should analyse the school environment factors for efficient assessment and evaluation processes.

## 5. Conclusions

The negative, widespread effects of EFD on occupational performance interfere with adolescents’ independence in occupations from self-care routines and social interactions to finishing homework and extend into the classroom. Consistently, adolescents with EF issues have been considered as struggling to start a task, understand what the task requires of them, realise they need, and then ask for, help and recognise when they do not have all the necessary information [9].

This study’s findings add to the theoretical and practical evidence of components that can assist and improve participation for adolescents both with and without EFD in general and at home specifically. From the users’ viewpoint, supportive school environments may include, for example, physically organising the classroom, providing quiet work areas for children who are distracted by various environmental stimuli, establishing small work groups and dividing tasks into stages with increasing levels of difficulty (to give the children a sense of success and motivation for tasks at higher challenge levels). The emphasis should be on allowing the children to acquire self-management skills in academic and day-to-day tasks (work on problem-solving, planning and, especially, control abilities) and adapting the children’s abilities (i.e., allowing the children to recognise their strengths and abilities and understand their difficulties). School procedures can be modified to provide relevant adjustments for each child, to be in constant contact with the children’s parents and to envision the children and their needs beyond the school framework.

Improved daily activities performance by adolescents with or without EFD can be possible through involvement in a supportive school environment. Assessing the adolescents’ daily activities performance can help determine their level of independence in performing everyday activities. It can educate the entire interdisciplinary team, caregivers and families for optimal intervention, discharge coordination and long-term planning.

## Figures and Tables

**Table 1:** Means, standard deviations and correlations among study variables.

Variable	M	SD	1	2	3	4	5	6	7	8	9	10
Frequency (home)	5.91	0.57										
2. Involvement (home)	3.79	1.04	0.45 ***									
3. Frequency (school)	4.52	1.33	0.29 **	0.11								
4. Involvement (school)	3.47	1.34	0.34 **	0.54 ***	0.67 ***							
5. Frequency (community)	3.92	1.11	0.15	0.35 **	0.30 **	0.36 **						
6. Involvement (community)	3.52	1.16	0.26 *	0.69 ***	0.09	0.42 ***	0.74 ***					
7. Support (Home)	9.31	2.50	0.38 ***	0.37 **	0.25 *	0.38 **	0.23 *	0.28 *				
8. Support (School)	13.16	3.25	0.40 ***	0.36 **	0.38 ***	0.40 ***	0.10	0.17	0.68 ***			
9. Support (Community)	12.85	2.96	0.27 *	0.39 ***	0.17	0.36 **	0.18	0.28 *	0.58 ***	0.65 ***		
10. Daily functioning	3.40	0.51	0.40 **	0.46 **	0.22 *	0.43 **	0.27 *	0.34 **	0.55 ***	0.72 ***	0.59 ***	
11. Functioning compared to peers	3.60	1.02	0.44 **	0.39 **	0.39 **	0.48 **	0.28 *	0.30 **	0.55 ***	0.72 ***	0.53 ***	0.88 ***

Note. * *p* < 0.05, ** *p* < 0.01, *** *p* < 0.001.

**Table 2:** Model coefficients.

Latent and Observed Variable			β	*p*
Daily activities performance	<---	Support (school)	0.609	***
Daily activities performance	<---	Support (home)	0.064	0.547
Daily activities performance	<---	Support (community)	0.151	0.156
Home	<---	Daily activities performance	0.531	0.016
School	<---	Daily activities performance	0.061	0.398
Community	<---	Daily activities performance	0.262	0.061
School	<---	Support (school)	−0.978	0.879
Home	<---	Support (home)	0.292	0.099
School	<---	Support (community)	0.040	0.803
Home	<---	Support (school)	−0.063	0.778
Community	<---	Support (school)	−0.126	0.702
School	<---	Support (home)	0.424	0.878
Community	<---	Support (home)	0.144	0.395
Home	<---	Support (community)	0.113	0.512
School	<---	Support (community)	0.395	0.897
Frequency (home)	<---	Home	0.594	
Involvement (home)	<---	Home	0.644	***
Frequency (school)	<---	School	1.664	
Involvement (school)	<---	School	7.697	0.331
Frequency (community)	<---	Community	0.625	
Involvement (community)	<---	Community	1.190	***
Daily functioning	<---	Daily activities performance	0.945	
Functioning compared to peers	<---	Daily activities performance	0.935	***

Note. *** *p* < 0.001.

## Data Availability

The data presented in this study are available on request from the corresponding author. The data are not publicly available due to ethical restrictions.
